# Performance of maximum likelihood mixture models to estimate nursery habitat contributions to fish stocks: a case study on sea bream *Sparus aurata*

**DOI:** 10.7717/peerj.2415

**Published:** 2016-10-04

**Authors:** Edwin J. Niklitschek, Audrey M. Darnaude

**Affiliations:** 1 Centro i∼mar, Universidad de Los Lagos, Puerto Montt, Los Lagos, Chile; 2 Center for Marine Biodiversity, Exploitation & Conservation, Centre National de la Recherche Scientifique, Montpellier, France

**Keywords:** Otolith chemistry, Mixture models, Mixed stocks, Mixing proportions, Mixing models, Stock identification, Stock structure, Fish stocks, Population structure, *Sparus aurata*

## Abstract

**Background:**

Mixture models (MM) can be used to describe mixed stocks considering three sets of parameters: the total number of contributing sources, their chemical baseline signatures and their mixing proportions. When all nursery sources have been previously identified and sampled for juvenile fish to produce baseline nursery-signatures, mixing proportions are the only unknown set of parameters to be estimated from the mixed-stock data. Otherwise, the number of sources, as well as some/all nursery-signatures may need to be also estimated from the mixed-stock data. Our goal was to assess bias and uncertainty in these MM parameters when estimated using unconditional maximum likelihood approaches (ML-MM), under several incomplete sampling and nursery-signature separation scenarios.

**Methods:**

We used a comprehensive dataset containing otolith elemental signatures of 301 juvenile *Sparus aurata*, sampled in three contrasting years (2008, 2010, 2011), from four distinct nursery habitats. (Mediterranean lagoons) Artificial nursery-source and mixed-stock datasets were produced considering: five different sampling scenarios where 0–4 lagoons were excluded from the nursery-source dataset and six nursery-signature separation scenarios that simulated data separated 0.5, 1.5, 2.5, 3.5, 4.5 and 5.5 standard deviations among nursery-signature centroids. Bias (*BI*) and uncertainty (*SE*) were computed to assess reliability for each of the three sets of MM parameters.

**Results:**

Both bias and uncertainty in mixing proportion estimates were low (*BI* ≤ 0.14, *SE* ≤ 0.06) when all nursery-sources were sampled but exhibited large variability among cohorts and increased with the number of non-sampled sources up to *BI* = 0.24 and *SE* = 0.11. Bias and variability in baseline signature estimates also increased with the number of non-sampled sources, but tended to be less biased, and more uncertain than mixing proportion ones, across all sampling scenarios (*BI* < 0.13, *SE* < 0.29). Increasing separation among nursery signatures improved reliability of mixing proportion estimates, but lead to non-linear responses in baseline signature parameters. Low uncertainty, but a consistent underestimation bias affected the estimated number of nursery sources, across all incomplete sampling scenarios.

**Discussion:**

ML-MM produced reliable estimates of mixing proportions and nursery-signatures under an important range of incomplete sampling and nursery-signature separation scenarios. This method failed, however, in estimating the true number of nursery sources, reflecting a pervasive issue affecting mixture models, within and beyond the ML framework. Large differences in bias and uncertainty found among cohorts were linked to differences in separation of chemical signatures among nursery habitats. Simulation approaches, such as those presented here, could be useful to evaluate sensitivity of MM results to separation and variability in nursery-signatures for other species, habitats or cohorts.

## Introduction

Evaluating the contribution of different sources to a mixture is a common problem in ecology, biology and natural resource management (Kimura & Chikuni, 1987; Smouse, Waples & Tworek, 1990; Van Dongen, Lens & Molemberghs, 1999; Fleischman & Burwen, 2003; Manel, Gaggiotti & Waples, 2005; Phillips, Newsome & Gregg, 2005; Newman & Leicht, 2007). Fish ecologists and fisheries scientists, for example, are frequently interested in estimating the contribution from different nursery habitats (sources) to adult aggregations, demographic units or stocks (mixtures). Beyond its inherent scientific interest, this kind of assessment has practical relevance for both management and conservation purposes (Kerr, Cadrin & Secor, 2010). Assessing the accuracy and precision of parameters resulting from mixture analysis is a fundamental, still often neglected step, required to facilitate the incorporation of these results into modern management models (Kritzer & Liu, 2014).

Mixture analysis in fish ecology and other disciplines relies heavily on the use of artificial and natural tags suitable for tracking or identifying the different sources (origins) contributing to a mixture (Gillanders, 2009). Within natural tags, the elemental and isotopic composition of teleost fish otoliths has been an increasingly common choice for this type of studies during the last decades (Kerr & Campana, 2014). Otoliths grow throughout lifetime by a regular deposition of calcium carbonate and protein layers, which, unlike bones, are not reabsorbed (Panfili et al., 2002). While calcium can be partially replaced by other metals (including Sr, Mn and Ba), dominant carbon and oxygen isotopes (^12^C and ^16^O) can be replaced by their less frequent alternatives ^13^C and ^18^O. When these substitutions are under weak internal control, they may reflect environmental and/or physiological variability (Panfili et al., 2002), and the elemental/isotopic otolith signatures can be considered “fingerprints” for the water masses inhabited by fish at carbonate deposition time (Elsdon et al., 2008). As deposition time can be often inferred from the same otolith through ageing techniques, a retrospective identification of nursery or feeding habitats, demographic units (∼stocks) and/or migration patterns becomes possible (Campana & Thorrold, 2001; Rooker & Secor, 2004; Elsdon et al., 2008; Rachel et al., 2008; Arkhipkin, Schuchert & Danyushevsky, 2009; Darnaude et al., 2014; Niklitschek et al., 2014).

Two main statistical approaches have been used to estimate the contribution of different sources to a mixture: Discriminant Functions (DF) and Mixture Models (MM) (Millar, 1990a; Koljonen, Pella & Masuda, 2005). DF approaches include linear discriminant analysis (LDA), quadratic discriminant analysis (QDA), multinomial regression (MNR) and random forest analysis (RM), among several other related methods (Edmonds, Caputi & Morita, 1991; Elsdon & Gillanders, 2003; Pella & Masuda, 2005; Mercier et al., 2011; Jones, Palmer & Schaffler, 2016). Although some parametric DF can be seen as special cases of MM, they have some important differences in focus and estimation procedures. DF focus on developing discriminant algorithms, which are fit (“trained”) using samples from known origins (i.e. pre-migratory juveniles sampled at their nursery-sources), and applied, at a subsequent step, to assign putative origins to older (adult) individuals sampled from the mixed-stock. Therefore, mixing proportions are not estimated directly as model parameters, but quantities computed afterwards from the putative origins assigned by the model to the individuals present in the mixed-stock dataset. MM approaches focus, instead, on estimating these mixing proportions, which are explicit and fundamental model parameters, estimated directly from the mixed-stock dataset. Baseline nursery-signatures are also explicit MM parameters, which are commonly of high scientific interest on their own.

Described in detail by Everitt & Hand (1981), MM were probably introduced into fisheries science by Cassie (1954). Applications to mixed stock analysis were first presented by Fournier et al. (1984) and increased largely after the HISEA software was made available by Millar (1990b). Under their finite mixture distribution form (Everitt & Hand, 1981), MM are defined as,

$$f(x) = \mathop \sum \limits_{k{\rm{ = 1}}}^K {p_k} \cdot g\left( {{x_i};{\theta _k}} \right)$$

where, the density function *f(x)* is defined by three groups of parameters: the number of components or sources (*K*), the mixing proportions (*p_k_*) and the set of baseline parameters *θ_k_* that characterize each source *k,* given the probability distribution function *g*(). This function is frequently, although not necessarily, assumed multivariate normal. Thus, *θ_k_* can be decomposed in a vector of means (μ*_k_*) and a covariance matrix (Σ_*k*_) for all response variables used to characterize each source *k*. Translating this terms into the lexicons of otolith chemistry and mixed-stock analysis, *K* corresponds to the number of nursery or spawning sources, *p_k_* to the proportional contribution made by each of these sources to the mixed stock, and *θ_k_* to the baseline chemical signatures (i.e. means and covariances of elemental or isotopic ratios) that characterize otolith material formed at each nursery source *k*.

Most MM applications to fisheries during the last four decades have used maximum likelihood methods (Millar, 1987; Reynolds & Templin, 2004). Within this framework, the expectation-maximization algorithm (EM) (Dempster, Laird & Rubin, 1977) has been used as the dominant likelihood maximization procedure. In more recent years, and following an evident worldwide trend in statistical methods, an important development of Bayesian approaches has been reflected in an increasing number of mixed stock applications (Pella & Masuda, 2001; Koljonen, Pella & Masuda, 2005; Munch & Clarke, 2008; White et al., 2008; Smith & Campana, 2010; Standish, White & Warner, 2011), including parametric and non-parametric approaches and important software development efforts (Neubauer, Shima & Swearer, 2013). Despite of these promising developments, MM methods probably remain as the most common approach being used for stock mixture analysis at scientific and management organizations.

Most MM applications to mixed stock analysis tend to focus on estimating *p_k_*, given all potential nursery sources have been previously identified (i.e. *K* is known) and sampled for pre-migratory juveniles to produce *ex-ante θ_k_* estimates, which are then supplied to the MM as fixed quantities. This conditional MM approach follows Millar (1987) and tends to be dominant in the MM literature (Hamer, Jenkins & Gillanders, 2005; Crook & Gillanders, 2006; Schloesser et al., 2010; Secor, Gahagan & Rooker, 2012). Some drawbacks of this approach, shared by DF methods, are that it fails if not all nursery-sources are known or sampled, and that it neglects all information about *θ_k_* being contained in the mixed-stock data. Under an unconditional MM approach, ${\hat \theta _k}$ are produced or updated using the information contained in the mixed-stock data. Thus, unconditional models benefit (greatly) from nursery-source sampling, but can still be fit if such sampling fails for some or all nursery-sources. Moreover, these models are expected to be less sensitive to small sampling sizes (Koljonen, Pella & Masuda, 2005).

Failing to sample some or all nursery-sources is a common problem in fish ecology, particularly for open marine populations (Campana et al., 2000), where the location of nursery habitats may be unknown, remote or inaccessible, or where juvenile fish may be to cryptic or invulnerable to the sampling gear. Under these scenarios, there may not be other option than the simultaneous (unconditional) estimation of both *p_k_* and *θ_k_* parameters from the mixed-data (Smouse, Waples & Tworek, 1990; Niklitschek et al., 2010; Smith & Campana, 2010). Furthermore, if not even *K* (the total number of nursery-sources) is known, all three sets of parameters (*p_k_*, *θ_k_* and *K*) need to be estimated from the mixed-stock data. Estimating all three sets of parameters within the same likelihood maximization fit may lead however to identifiability issues. As an alternative, a model comparison approach can be used (Everitt & Hand, 1981), to select the most informative value of *K* according to some criterion such as Akaike (1973) and Schwarz (1978), entropy (Celeux & Soromenho, 1996), deviance (White et al., 2008) or some other information criterion, depending on the modelling framework being used.

The simultaneous estimations of *p_k_*, *θ_k_* and/or *K* from the mixed-stock data might introduce bias related to the existence of multiple solutions and/or to multiple local maxima (McLachlan & Peel, 2004; Reynolds & Templin, 2004), as well as to the presence of confounding covariates affecting the mixed-stock data, which may either blur or spuriously enhance variability in nursery signatures. Among the three sets of MM parameters, *K* seems to be the most prone to bias, as shown by several theoretical and practical studies (Titterington, 1990; Celeux & Soromenho, 1996; White et al., 2008; Neubauer, Shima & Swearer, 2013). Assessing the magnitude of such bias under incomplete sampling scenarios is not frequently reported (Wood et al., 1987) as no reference data exists to contrast the parameters estimated by the model. Indirect assessment approaches can be conducted, however, using either simulated or empirical datasets whose true *p_k_*, *θ_k_* and/or *K* parameters were actually known.

In this article, we evaluate the performance of maximum likelihood mixture models, from now on “ML-MM,” to estimate *p_k_*, *θ_k_* and *K* parameters under several scenarios that simulated incomplete sampling and different degrees of separation among nursery signatures. Departing from the mainstream of ML-MM, we adopted an unconditional approach to estimate and/or update ${\hat \theta _k}$ using all available nursery-source and mixed-stock data. To conduct this evaluation, we follow a case study approach focused on a comprehensive spatio-temporal dataset containing baseline chemical signatures from young-of-the-year *Sparus aurata* collected in four separate nursery habitats (Mediterranean lagoons), in three highly contrasting years (Tournois et al., 2013). By sub-setting, resampling and manipulating this dataset we evaluated bias and uncertainty in *p_k_*, *θ_k_* and *K* as a function of (i) the number of nursery sources identified and sampled for pre-migratory juveniles to estimate nursery-signature parameters, (ii) the observed variability in nursery-signatures among cohorts, and (iii) the degree of separation among nursery-signature centroids.

## Materials and Methods

### Data set description

The dataset used in the present work, obtained from Tournois et al. (2013), included otolith fingerprints from 301 young-of-the-year *Sparus aurata,* collected in the Gulf of Lions (NE Mediterranean Sea) from four Mediterranean lagoons: Bages-Sigean, Mauguio, Salses-Leucate and Thau, in three different years (= cohorts): 2008, 2010, and 2011. Collection always occurred in the late summer, just before they returned to mix with individuals from nearby lagoons in the open sea.

The chemical composition analysis of otolith samples was performed using Solution Based Inductively Coupled Plasma Mass Spectrometry, and included ^43^Ca and another 11 elements (Tournois et al., 2013). For this study, we only kept the seven most discriminant ones: ^7^Li, ^11^B, ^25^Mg, ^85^Rb, ^86^Sr, ^89^Y and ^138^Ba. Their concentrations in the otoliths were expressed as elemental ratios to Ca, and standardized to mean = 0, and SD = 1 to scale all elements equally and facilitate bias analysis. Three obvious outliers were discarded, working with a depurated sample size of 298 otoliths. Data was normalized using a multivariate Box & Cox (1964)’s transformation although it failed to fully normalize three of the seven elemental ratios (Mg, Rb and Ba).

The four lagoons sampled by Tournois et al. (2013) differ greatly in size, depth, freshwater input and degree of connection with the sea, leading to physical and chemical differences in the water and, therefore, in otolith signatures of juvenile *S. aurata* (Tournois et al., 2013). Nonetheless, these lagoons are strongly influenced by rainfall, wind and other environmental forces (Sara, Leonardi & Mazzola, 1999; Martins et al., 2001), leading to high interanual variability in otolith signatures (descriptive statistics provided in Appendix 2). For instance, the average squared Mahalanobis distance among nursery sources decreased from 3.29 to 1.18 SD, between 2008 and 2011, while its multivariate spread (effective general variance) ranged between 0.54–0.86 within the same interval (Table 1). A multivariate analysis of variance showed significant effects of cohort, nursery-source and their interaction upon these nursery signatures (p < 0.001).

**Table 1:** Average Mahalanobis distance and effective standard deviation for elemental compositions of selected metals in otoliths of juvenile *Sparus aurata*. Average Mahalanobis distance and effective standard deviation for elemental compositions of selected metals in otoliths of juvenile *Sparus aurata*. Average distances within nursery source and cohort computed from vectors of observations. Average distances within years and within sources computed from vectors of means corresponding to each source or year, respectively. All values computed after standardizing all data to = 0 and = 1.

Cohort	Bages-Sigean	Mauguio	Salses-Leucate	Thau	Within cohorts
Average squared Mahalanobis distance (Δ^2^)					
2008	2.52	2.53	2.53	2.55	3.29
2010	2.47	2.52	2.47	2.49	2.78
2011	2.48	2.54	2.51	2.50	1.18
Within sources	3.49	3.21	2.20	1.88	3.50
Effective standard deviation |S|^1/14^					
2008	0.40	0.39	0.35	0.40	0.65
2010	0.41	0.51	0.63	0.53	0.86
2011	0.38	0.39	0.46	0.49	0.54
Within sources	0.63	0.66	0.73	0.71	0.85

### Simulation approach and scenarios

All simulations and analyses were based upon the construction of two datasets: (i) a “nursery-source dataset” that represented otolith composition data from pre-migratory juveniles, sampled at their corresponding nursery-origins, and (ii) a “mixed-stock dataset” that represented otolith composition data from older fish sampled after they had mixed with fish from the other four origins (i.e. at the open sea). Besides the observed variability among the three sampled cohorts, we simulated additional variability in two dimensions: (i) the number of sources being sampled and included in the “nursery-source dataset” and/or (ii) the degree of separation among nursery-signature centroids (Table 2).

**Table 2:** Main configuration of the simulation and resampling procedures used for assessing the performance of maximum likelihood mixed models. Main configuration of the simulation and resampling procedures used for assessing the performance of maximum likelihood mixed models. Observed cohorts corresponded to juvenile *Sparus aurata* collected from four Mediterranean lagoons in three highly contrasting years. Virtual cohorts corresponded to artificial data, aimed to expand the observed range of separation among nursery signatures *k* and *y* subscripts represent nursey-sources and cohorts, respectively. Observed mean vectors and covariance matrices available in Appendix 2.

	Observed cohorts	Virtual cohorts
Number of nursery sources included in nursery-source datasets (*K_S_*)	0–4	0–4
Number of cohorts	3	6
Nursery-signature mean vectors (μ_*k,y*_)	Observed *μ_k,y_*	*μ*_*k*,2010_ scaled to match target separation
Nursery-signature covariance matrices Σ*_k,y_*	Observed Σ*_k,y_*	Observed Σ_*k*,2010_
Average Mahalanobis distance among nursery-signature centroids $(\Sigma^2_{y})$	Observed $\Delta^2_{y}=1.18-3.29$	Simulated $\Delta _y^2$ = {0.5, 1.5, 2.5, 3.5, 4.5, 5.5}
Mixing proportion of nursery-sources in the mixed-stock dataset *p_k,y_*	*p_k,y_* = {0.1, 0,2, 0.3, 0.4}, randomly assigned to sources within runs	*p_k,y_* = {0.1, 0,2, 0.3, 0.4}, randomly assigned to sources within runs

#### Nursery-source sampling scenarios

Five scenarios were defined by the number of nursery-sources included in the nursery-source dataset. At each run, *K_S_* = 0–4 sources were randomly selected to be included in the nursery-source dataset as “known” nursery habitats, which had been sampled for pre-migratory juveniles. These data had been used as baseline data to produce initial estimates for ${\hat \theta _k}$ and then mixed with the mixed-stock data to produce final ${\hat \theta _k}$ parameters, following an unconditional likelihood maximization approach. All remaining “unknown” nursery sources (*K_U_ = K−K_S_*) were excluded from the nursery-source dataset and lacked of initial ${\hat \theta _k}$ values.

#### Separation among nursery signatures

To improve our empirical understanding about the effects the separation among nursery-signature centroids may have on bias and uncertainty in ML-MM parameters, we applied the five sampling scenarios to (i) the three observed cohorts (2008, 2010 and 2011); and (ii) six virtual cohorts created to expand the range of Mahalanobis distances Δ^2^ among nursery-signature centroids observed in the three sampled cohorts (Δ^2^ = 1.18–3.29), Table 1. To build these six virtual cohorts, covariance matrices were set equal to those estimated in 2010 for each nursery-source (Appendix 1), while the vectors of means correspondng to this same year (Appendix 1) were re-scaled to get Δ^2^ values of 0.5, 1.5, 2.5, 3.5, 4.5 and 5.5 (Table 2).

#### Resampling procedures

Datasets for each tested scenarios and independent run were produced by parametric bootstrapping. Nursery-source datasets included 25 observations drawn from each of the *K_S_* known nursery-sources and from each of the three cohorts. Mixed-stock datasets included a total of 100 observations per cohort, drawn from all four nursery-sources, mixed using uneven mixing proportions (*m*) of 0.1, 0.2, 0.3 or 0.4. These four proportions represented the true value of *p_k_* and were randomly allocated to the four nursery-sources, within each resampling run. Resampling was followed by a standard modelling and fitting procedure, detailed in Appendix 1. This was a 12-steps sequence, which was repeated 1,000 times for each cohort and scenario. Each repetition was labelled as a “resampling run.”

### Mixing proportions

To evaluate bias and uncertainty of mixing proportion estimates (${\hat p_k}$), we assumed the true number of nursery sources was known and fixed (*K* = 4) across all scenarios. Bias in $\hat p$ was computed as the sum of the average differences between the estimated (${\hat p_{mr}}$) and the true mixing proportion (*p_mr_*) assigned to each nursery-source within each resampling run. The subscript *m* = {0.1, 0.2, 0.3, 0.4} represents here the vector of mixing proportions randomly allocated to the four nursery-sources, within each of the R = 1,000 resampling runs. Therefore,

$$B{I_{\hat p}}{\rm\,{ = }}\sum\limits_{m = 1}^M {{1 \over R}{\rm{ }}\sum\limits_{r = 1}^R {\left| {{{\hat p}_{mr}}-{p_{mr}}} \right|} } $$

Uncertainty in ${\hat p_k}$ was indexed as the average of the four empirical standard errors of $\hat p$ computed for each of the four possible values of *m*, across the R = 1,000 resampling runs,

$${SE_{\hat p}}{\rm\,{ = }}{1 \over M}\sum\limits_{m = 1}^M {\sqrt {{{{{({{\hat p}_{mr}}-{p_{mr}})}^2}} \over R}} } $$

### Nursery-signature parameters

Under the assumption of multivariate normal distribution, each set of estimated nursery-signature parameters ${\hat \theta _k}$ was composed by a vector of means ${\hat \mu _k}$ and a covariance matrix ${\hat \Sigma _k}$, which described the multivariate distribution of the seven chemical elements measured in the otoliths included in the dataset. Assessing bias in ${\hat \Sigma _k}$ is a complex task, which we considered that exceeded the scope of this paper. Therefore, all bias measures provided hereafter for ${\hat \theta _k}$ are strictly referred to ${\hat \mu _k}$.

As done for ${\hat p_k}$, the assessment of bias and uncertainty in ${\hat \theta}$ was conducted assuming a known and fixed value of *K* = 4. Overall bias in ${\hat \theta _k}$ was indexed by averaging the absolute mean differences between estimated (${\hat \mu _{kr}}$) and true (*μ_kr_*) vectors of means, across all elemental ratios (*J* = 7) and nursery-sources (*K* = 4). As all elemental ratios were previously standardized, bias units were equivalent to standard deviations and computed as follows,

$$B{I_{\hat \theta }}{\rm\,{ \,=\, }}{1 \over {J \cdot K}}\mathop \sum \limits_{j = 1}^J \mathop \sum \limits_{k = 1}^K \left| {{1 \over R}\sum\limits_{r = 1}^R {{{\hat \mu }_{kr}}-{\mu _k}} } \right|$$

Overall uncertainty in ${\hat \theta _i}$, was indexed by its effective standard deviation (Peña & Rodríguez, 2003), defined as,

$${{S}}{{{E}}_{\hat \theta }}{\rm{ \,= \,}}{1 \over K}\sum _{k \,=\, 1}^K{\left| {{{\widehat \sum }_k}} \right|^{1/2J}}$$

where, ${\hat \Sigma _k}$ is the covariance matrix computed from all ${\hat \mu _{kr}}$ estimated for each nursery-source *k* across the R = 1,000 resampling runs.

### Number of contributing nursery sources

For assessing bias and uncertainty in $\hat K$, this parameter was not set to a constant as before, but estimated by a model selection approach as described in the next section. Bias in $\hat K$ was computed as $B{I_{\hat K}}{\rm{ \,=\, }}{\hat {\rm K}}-4$, and uncertainty ($S{E_{\hat K}}$) as the standard error of $\hat K$ computed from all resampling runs, within each cohort and scenario. The strength of the selection was indexed by $\Delta BI{C_K}$, defined as the average of the differences between the lowest and the median Bayesian Information Criterion (BIC) values computed for all competing models, within each resampling run.

### ML-MM parameter estimation

Parameters ${\hat p_k}$ and ${\hat \theta _k}$ (${\hat \mu _k}$ and ${\hat \Sigma _k}$) were estimated by maximum likelihood, using the Expectation-Maximization (EM) algorithm (Dempster, Laird & Rubin, 1977), modified to follow an unconditional approach where initial ${\hat \theta _k}$ estimates were updated to maximize the joint likelihood of both nursery-source and mixed-stock datasets (Appendix 1). The M-step was constrained to produce definite positive covariance matrices, with det(Σ) > 10^9^. Starting ${\hat \mu _k}$ and ${\hat \Sigma _k}$ parameters for the *K_S_* known nursery-sources were computed directly from the nursery-source dataset. Starting ${\hat \mu _k}$ for the *K_U_* unknown nursery-sources were obtained from the mixed-stock dataset trough a semi-supervised *K*-means clustering procedure, implemented using the R-package “vegclust” (De Cáceres, Font & Oliva, 2010), which allowed for combining “fixed” and “mobile” centroids. Fixed centroids corresponded to ${\hat \mu _k}$ estimated at the previous step for the *K_S_* known nursery-sources. *K_U_* additional mobile centroids, which represented the *K_U_* unknown nursery sources, were estimated as the values that minimized the mean square distance between the mixed-stock data and all (fixed and mobile) model centroids. Starting ${\hat \Sigma _k}$ for the *K_U_* unknown nursery-sources were computed, at a subsequent step, from the mixed-stock data clustered into each of these *K_U_* additional clusters (Appendix 1). Starting ${\hat p_k}$ were calculated as the empirical proportion of individuals represented in the mixed-stock dataset assigned to each putative nursery-source *k* in order to maximize de probability density of each observation $p({x_i}|{\hat \theta _k})$, assuming ${x_i} \sim {\rm{MVN(}}{\hat \theta _k}{\rm{)}}$.

Parameter $\hat K$ was estimated following a model selection procedure, where multiple competing models were fit to each pair of nursery-source and mixed-stock datasets. These competing models considered a range of *K* values, between a minimum of *K_min_* = *K_S_* and a maximum defined arbitrarily as *K_max_* = 8. As result, within each resampling run, and depending upon the value of *K_S_*, a total of 4–9 competing models were fit and compared. Model comparisons were performed using Schwarz (1978)’s BIC, where the most informative value of *K* was addressed as the “estimated” number or nursery-sources ($\hat K$).

## Results

### Mixing proportions

**Figure 1:**
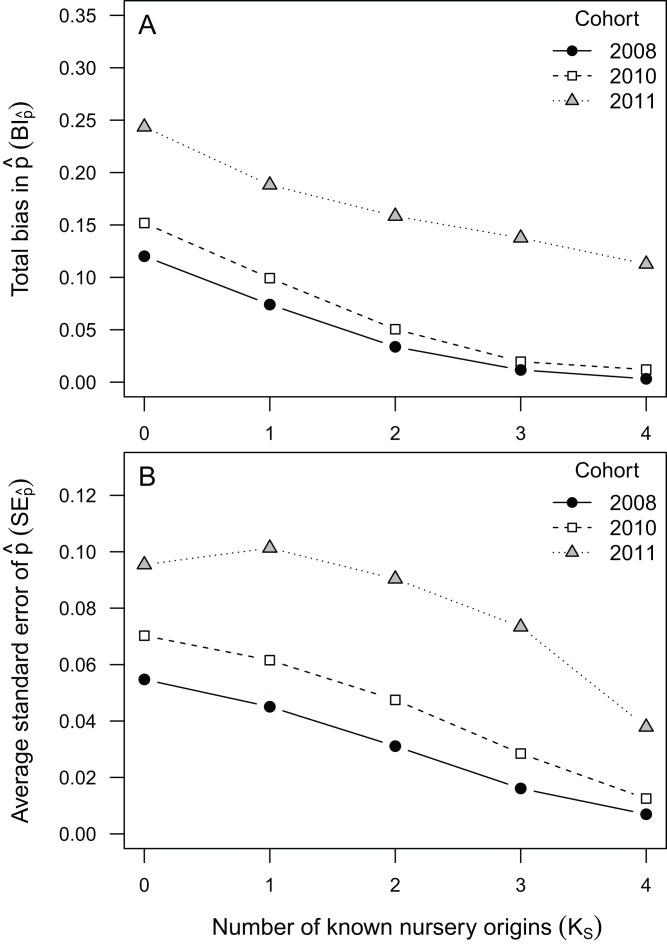
Bias and uncertainty in mixing proportions for observed cohorts. Bias (A) and uncertainty (B) in mixing proportions ($\hat p$) of four nursery-sources to artificial mixed-stocks of *Sparus aurata* built using data from cohorts 2008, 2010 and 2011. Different sampling scenarios are represented by the number of nursery-sources (*K_S_*) simulated to be known and sampled for pre-migratory juveniles.

Bias in $\hat p(B{I_{\hat p}})$ ranged between 0 and 0.24 across all data availability scenarios and observed cohorts. Relatively unbiased $\hat p$ estimates $(B{I_{\hat p}} < 0.1)$ were obtained under most data availability scenarios (*K_S_* = 1–4) for cohorts 2008 and 2010 (Table 3; Fig. 1), but exceeded 0.11 across all scenarios for cohort 2011. The highest values of $B{I_{\hat p}}$ (0.12–0.24) were found at *K_S_* = 0, when none of the nursery-sources were known (Table 3; Fig. 1). When all nursery-sources were known (*K_S_* = 4), $B{I_{\hat p}}$ approached zero for cohorts 2008 and 2010, but remained relatively high ($B{I_{\hat p}}$∼0.11) for cohort 2011. Such a decrease in bias was near one order of magnitude for cohorts 2008 and 2010, and greater than 50% for cohort 2011 (Fig. 1). Uncertainty in $\hat p(S{E_{\hat p}})$ ranged between 0.06 and 0.28, with much higher values observed for cohort 2011 ($S{E_{\hat p}}$ = 0.03–0.11) than for both cohort 2010 ($S{E_{\hat p}}$ = 0.007–0.05) and for cohort 2008 ($S{E_{\hat p}}$ = 0.01–0.07). Following a pattern somewhat similar to $B{I_{\hat p}}$, we found that $S{E_{\hat p}}$ decreased rapidly as *K_S_* increased (Fig. 1).

**Table 3:** True and estimated mixing proportions of nursery-sources in the mixed-stock dataset (*p_k_*). True and estimated mixing proportions of nursery-sources in the mixed-stock dataset (*p_k_*). Data from all nursery-sources combined for simplicity. True values corresponded to the proportion of bootstrap samples drawn from each cohort and nursery-source at each resampling runs (R = 1,000). These nursery-source proportions were assigned randomly from the vector *m* = {0.1, 0.2, 0.3, 0.4}.

Cohort	True proportion in mixed-stock dataset (*p_k_*)	Sampling scenario (number of habitats represented in nursery-source datasets)				
		*K_S_* = 4	*K_S_* = 3	*K_S_* = 2	*K_S_* = 1	*K_S_* = 0
2008	0.1	0.10	0.10	0.11	0.13	0.15
	0.2	0.20	0.20	0.20	0.21	0.21
	0.3	0.30	0.30	0.30	0.29	0.29
	0.4	0.40	0.40	0.39	0.37	0.35
2010	0.1	0.11	0.11	0.12	0.14	0.16
	0.2	0.20	0.20	0.21	0.21	0.22
	0.3	0.30	0.30	0.30	0.29	0.28
	0.4	0.40	0.39	0.38	0.36	0.35
2011	0.1	0.14	0.15	0.16	0.17	0.19
	0.2	0.21	0.22	0.22	0.23	0.23
	0.3	0.28	0.29	0.28	0.28	0.27
	0.4	0.36	0.34	0.34	0.33	0.31

**Figure 2:**
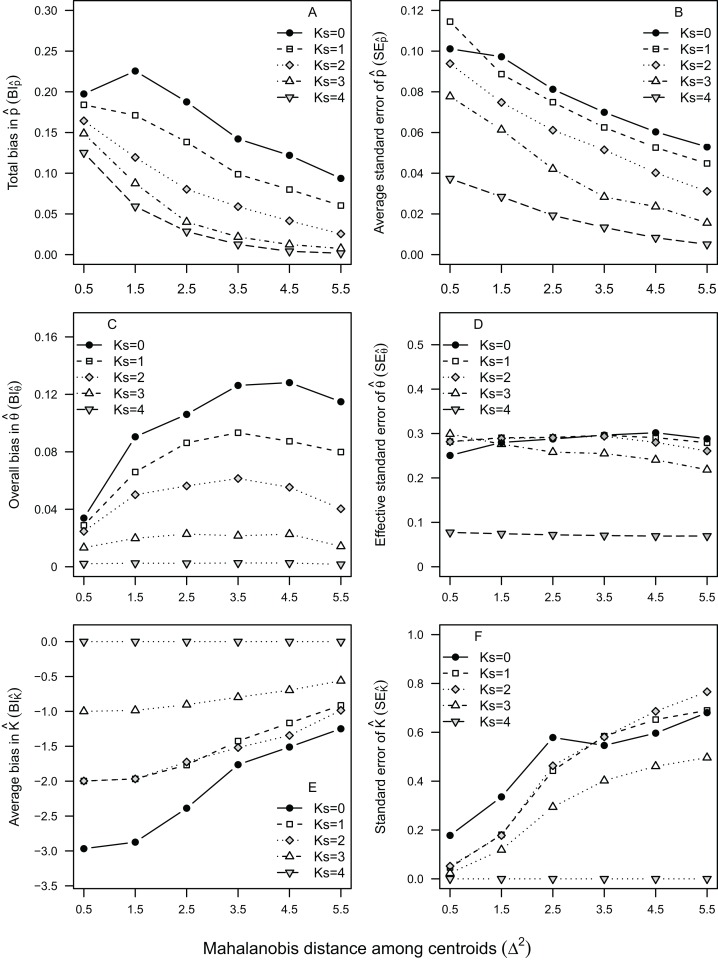
Bias and uncertainty in mixture model parameters for virtual cohorts of *Sparus aurata*. Average bias and uncertainty in mixing proportions $\hat p$ (A and B), nursery-signature estimates $\hat \theta$ (C and D) and number of contributing sources $\hat K$ (E and F) as a function of the simulated distance among nursery-signature signature centroids and the number of nursery-sources (*K_S_*) simulated to be known and sampled for pre-migratory juveniles.

The rank order of $B{I_{\hat p}}$ and $S{E_{\hat p}}$ values among the three observed cohorts was inverse to the rank order of their average distance among nursery-signature centroids (Table 1). This inverse relationship was also observed in the six nursery-signature separation scenarios, where $B{I_{\hat p}}$ and $S{E_{\hat p}}$ decreased rapidly as the distance among nursery signatures increased (Fig. 2). Such a decrease tended to evolve from a linear pattern at the worse 1–2 scenarios (*K_S_* = 0–1) to a more exponential decay pattern as *K_S_* approached its maximum (Fig. 2) There was also an evident trend to observe positive bias at lower $\hat p$ values, and negative bias at higher $\hat p$ values, which was more pronounced as *K_S_* decreased (Table 3).

### Nursery-signature parameters

**Figure 3:**
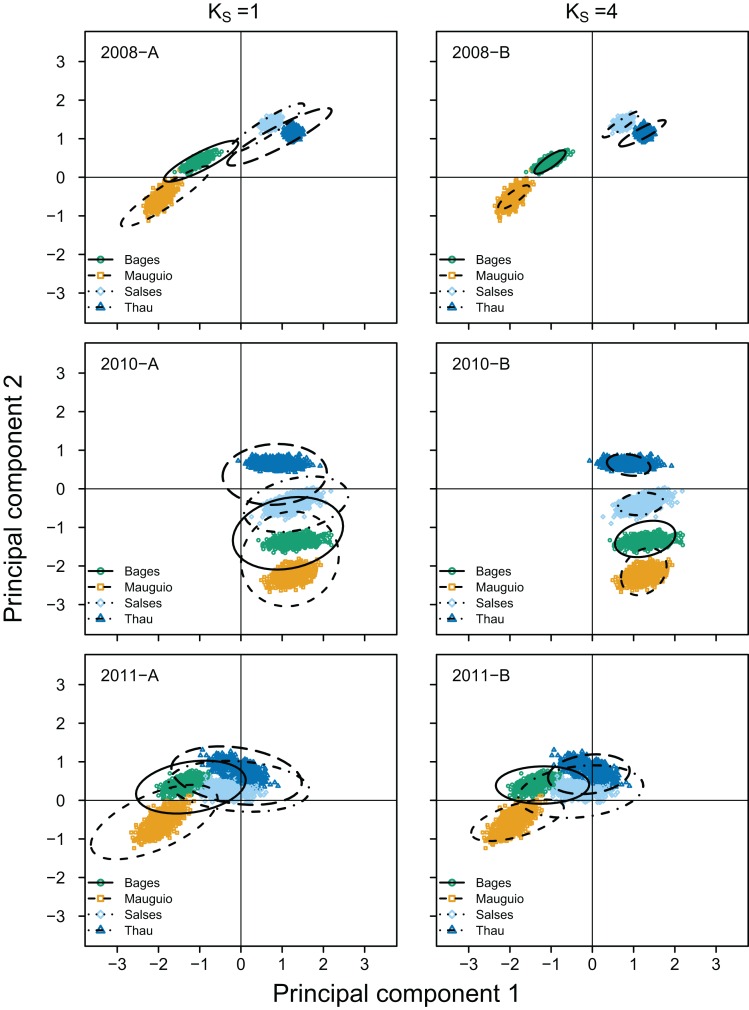
Empirical and estimated distributions of elemental ratios in juvenile *Sparus aurata* otoliths. Principal component diagrams representing the distribution of otolith elemental ratios in juvenile *Sparus aurata*. Coloured points represent empirical means per nursery source and cohort (2008, 2010 and 2011), corresponding to 1,000 bootstrap samples (size = 25). Ellipses represent 67% confidence intervals for estimated means under two selected sampling scenarios: *K_S_* = 0, where no nursery-sources were sampled for pre-migratory juveniles (2008-A, 2010-A and 2011-A) and *K_S_* = 3, where three out of the four nursery-sources were simulated as known and sampled for pre-migratory juveniles (2008-A, 2010-A and 2011-A).

**Figure 4:**
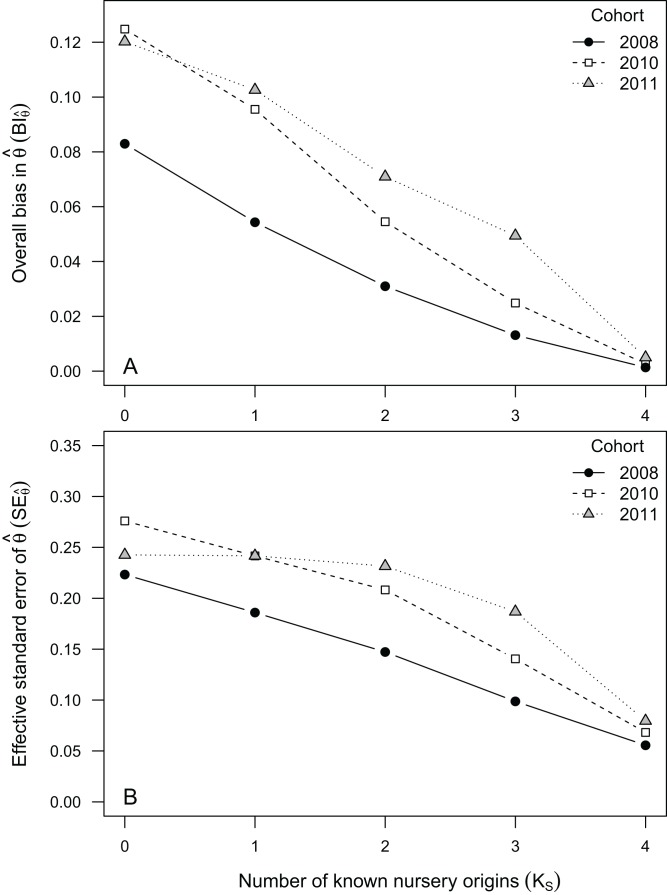
Bias and uncertainty in nursery-signature estimates. Average bias (A) and uncertainty (B) in estimated nursery signatures corresponding to juvenile *Sparus aurata* sampled from four nursery-sources in three climatically contrasting years, bootstrapped and combined into artificial mixed-stocks. Different sampling scenarios were represented by the number of nursery-sources (*K_S_*) simulated to be known and sampled for pre-migratory juveniles. Data obtained after 1,000 resampling runs per scenario.

Estimated nursery-signature parameters provided relatively unbiased and consistent (similar shape and orientation) fits to the “true” distribution of means, even at the *K_S_* = 0 scenario (Fig. 3). Considering the observed cohorts, $B{I_{\hat \theta }}$ ranged between 0.005 and 0.13 at *K_S_* = 4 and *K_S_* = 0, respectively (Fig. 4). As observed before for mixing proportions, $B{I_{\hat \theta }}$ tended to be much higher for cohort 2011 than for cohort 2008, across all scenarios. However, the values of $B{I_{\hat \theta }}$ for cohort 2010 tended to be much closer to those computed for cohort 2011, than to the ones computed for cohort 2008 (Fig. 4). As for the six nursery-signature separation scenarios (Fig. 2), $B{I_{\hat \theta }}$ tended to increase with distance from minimum values at Σ^2^ = 0.5 towards maximum values and highest sensitivity to incomplete sampling Δ^2^ values of 3.5 or 4.5 depending on the number of known nursery sources (Fig. 2).

Uncertainty in ${\hat \theta _k}(S{E_{\hat \theta }})$ was less sensitive to data availability (*K_S_*) than $B{I_{\hat \theta }}$, showing a moderate although nearly constant decrease with *K_S_*, in cohorts 2008 and 2010. For cohort 2011, however, $S{E_{\hat \theta }}$ was similarly high between *K_S_* = 0 and *K_S_* = 2, decreasing afterwards. Uncertainty in ${\hat \theta _k}$ tended to be higher for cohorts 2010 and 2011 than for cohort 2008, across all scenarios, but particularly between *K_S_* = 1 and *K_S_* = 3. Results from simulated nursery-signature separation scenarios (Fig. 2) showed $S{E_{\hat \theta }}$ was not affected by distance among nursery signatures when all nursery-sources were previously known and sampled (*K_S_* = 4). Otherwise, $S{E_{\hat \theta }}$ tended to decrease with distance among nursery signatures, although for *K_S_* = 0 this decrease was only evident at the two highest distances among nursery signatures (Fig. 2).

### Number of contributing nursery sources

**Figure 5:**
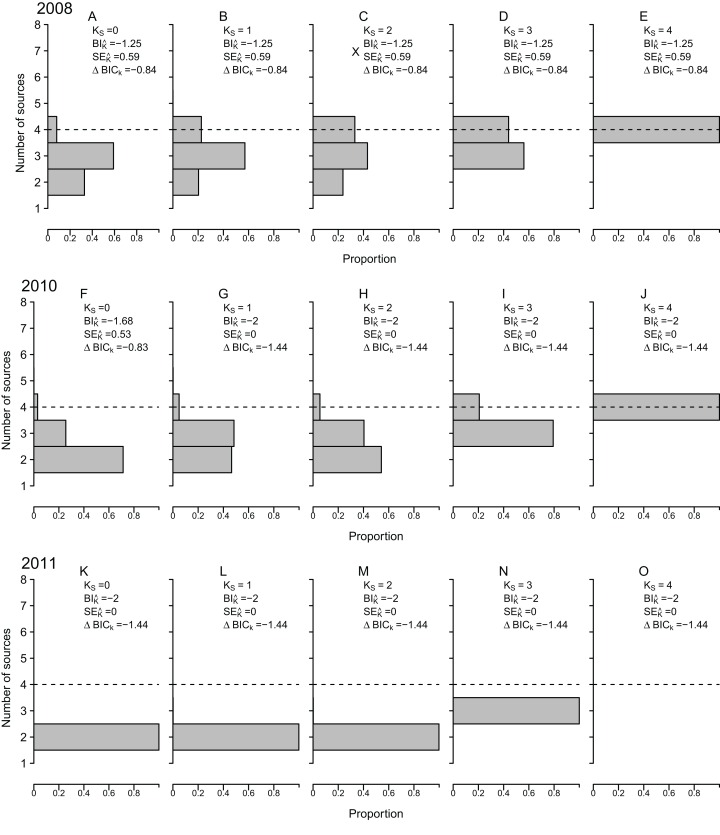
Estimated number of contributing sources. Estimated number of nursery-sources ($\hat K$) contributing to simulated mixed-stock of *Sparus aurata* obtained following a model selection approach based on Bayesian Selection Criterion. Different sampling scenarios were represented by the number of nursery-sources (*K_S_*) simulated to be known and sampled for pre-migratory juveniles. Relative frequencies computed after 1,000 resampling runs per tested scenario. $B{I_{\hat K}}$ = mean bias for $\hat K$; $S{E_{\hat K}}$ = mean standard error of $\hat K$; $\Delta BI{C_K}$ = difference between minimum and median values of the Bayesian Information Criterion.

ML-MM tended to underestimate the true value of *K* for all observed cohorts, under most data availability scenarios (Fig. 5), with negative bias ($B{I_{\hat k}}$), between −0.56 and −2, across all incomplete sampling scenarios. Only under the ideal scenario (*K_S_ = 4*) $B{I_{\hat k}}$ became zero and the true value of *K* was correctly estimated in 100% of all simulations. Nonetheless, it must be recalled that under *K_S_* = 4, $\hat K$ was constrained to values greater or equal to *K_min_* = 4. As for the remaining scenarios (*K_S_* < 4), the absolute value of $B{I_{\hat k}}$ tended to decrease as *K_S_* increased, at least for cohorts 2008 and 2010 (Fig. 5).

The under-estimation of $\hat K$ was a highly consistent pattern, observed across all nursery-signature separation scenarios, were no estimated values of $\hat K$ ≥ 5 were ever obtained, regardless of the data availability scenario or cohort (Fig. 2). Nonetheless, the magnitude of this bias decreased by 50–70% as the distance among nursery signatures increased (Fig. 2). Uncertainty in $\hat K$($S{E_{\hat K}}$) was zero at *K_S_* = 4, but relatively high ($S{E_{\hat K}}$ > 0.4) in cohorts 2008 and 2010 for all *K_S_* < 4, reaching maximum values at *K_S_* = 2 (Fig. 5). The strength of the model selection measured by $\Delta BI{C_K}$ tended to increase towards *K_S_* = 4, where it was maximum for cohorts 2008 and 2010 (Fig. 5). The consistent, although biased, values of $\hat K$($B{I_{\hat K}}$ = 1–2) lead to particularly low values of $S{E_{\hat K}}$ for cohort 2011, across all scenarios. Consistent values of $S{E_{\hat K}}$ ≈ 0 at *K_S_* = 4, and a weak relationship between *K_S_* and $S{E_{\hat K}}$ for all *K_S_* < 4 were observed across all nursery-signature separation scenarios. Nonetheless, it was evident that $S{E_{\hat K}}$ tended to increase with distance among nursery signatures for all incomplete sampling scenarios.

## Discussion

In this article, we combined real-world and virtual datasets to evaluate the performance of unconditional ML-MM when used to estimate the three fundamental sets of mixed stock parameters (*p_k_*, *θ_k_* and *K*), under a range of data availability and distance among nursery-signature scenarios. Although using a single real-world dataset might limit the generalization of our results, which may not be transferable to other stocks, we believe the large variability observed among cohorts in the real-world dataset, along with the additional variability included in the simulated datasets might represent a relevant part of the variability that could be found in other populations and geographical areas.

Mixing proportions estimated by ML-MM (${\hat p_k}$) showed low bias and variability when at least one nursery source was included in the nursery-source dataset. Large variability in bias and uncertainty of ${\hat p_k}$ was found, however, among cohorts, with cohort 2011 exhibiting the highest bias and uncertainty values in ${\hat p_k}$. This variability in bias and uncertainty among cohorts was consistent with inter-annual differences in sensitivity to incomplete sampling resulting from variable degrees of separation among nursery signatures. Results from simulations showed, for instance, that a minimum of three sampled nursery sources had been required to reduce $B{I_{\hat p}}$ below 0.1, given a Mahalanobis distance among nursery-signature centroids ≤ 1.5.

Overall our results confirmed the suitability of using ML-MM for estimating unbiased mixing proportions, given the number of nursery-sources (*K*) was known and there was a proper balance between the number of nursery-sources sampled for prior nursery-signature parameters and the actual degree of separation among nursery-signature centroids. Although results from our simulated separation scenarios cannot be turn into a prescriptive guideline, they explore such trade-offs and provide a general idea about which combinations might work, at least for Mediterranean stocks of *S. aurata*.

When incomplete sampling and/or reduced distance among nursery signatures biased mixing proportions estimates, bias tended to be greater for the most extreme ${\hat p_k}$ values, which were shifted towards intermediate values. Therefore, the smallest nursery contributions tended to be overestimated, while the largest ones to be underestimated. This behaviour is probably related to the model constrain that all proportions must sum one, which limits the parameter space and tend to increase negative correlation between the two most extreme values. It might be also somewhat related to the EM algorithm, which may converge to unsatisfactory local maxima (Marin, Mengersen & Robert, 2005).

Nursery-signature parameter estimates were relatively unbiased for all evaluated cohorts, under most data availability scenarios. When two or more nursery-sources were known and previously sampled, $B{I_{\hat \theta }}$ dropped below 0.10. Uncertainty in ${\hat \theta _k}$, however, was relatively high under incomplete sampling scenarios, particularly in cohorts 2010 and 2011, where $S{E_{\hat \theta }}$ exceeded 0.20 at all *K_S_* < 3 scenarios. The important differences in bias and uncertainty we found among cohorts, were likely related to large environmental variability in the study area (Tournois et al., 2013), which were reflected in highly variable distribution patterns of nursery signatures among cohorts (Fig. 3). Simulated nursery-signature separation scenarios showed a dome-shaped relationship between $B{I_{\hat \theta }}$ and distance among nursery-signature centroids, with maximum bias at intermediate distances and minimum bias at the shortest distance we tested (Δ^2^ = 0.5). This counter-intuitive pattern may have emerged from the antagonistic effects of less separable nursery signatures but more constrained parameter spaces at shorter distances among their centroids.

Unlike what we found for ${\hat p_k}$ and ${\hat \theta _k}$, the performance of our model selection approach for estimating the true number of nursery-sources (*K*) was poor and exhibited an evident trend to underestimate the true value of *K* by 1 or 2 nursery-sources in all observed and most of the simulated cohorts. As the magnitude of this underestimation bias was constrained by the number of known nursery-sources, it resulted obvious that enhancing our knowledge about the minimum number of contributing sources had reduced the risk of underestimating *K*. Given this biasing trend, the model selection approach we followed provided a lower bound rather than an accurate estimate of *K*.

Exploratory comparisons (not shown in results) between BIC and Akaike (1973)’s Information Criteria (AIC) yielded results that agreed with the idea that BIC produces underestimated but less variable estimates of *K*, while the opposite would be true for AIC (Koehler & Murphree, 1988). Alternative model selection criteria have been proposed by several authors and deserve further testing under a greater range of scenarios (Celeux & Soromenho, 1996). Departing from model selection approaches, bootstrapping has been proposed and used to evaluate consistency in $\hat K$ (McLachlan & Peel, 2004). Neubauer, Shima & Swearer (2013) followed, instead, a Bayesian approach to internalize the estimation of *K* into a Dirichlet process mixture model, which had the advantage of producing marginal distributions over a range of plausible $\hat K$ values and allowed for a direct probabilistic interpretation of model results. There is, however, a generalized view that estimating *K* is one of the most difficult tasks within MM, for which more satisfactory solutions are still needed (Celeux & Soromenho, 1996; McLachlan & Peel, 2004; White et al., 2008; Neubauer, Shima & Swearer, 2013).

Although we focused on testing the performance of unconditional ML-MM, we must acknowledge the growing importance of Bayesian approaches, observed in relatively recent years (Pella & Masuda, 2001; Marin, Mengersen & Robert, 2005; Munch & Clarke, 2008; White et al., 2008; Smith & Campana, 2010; Standish, White & Warner, 2011; Neubauer, Shima & Swearer, 2013). They represent obvious alternatives to conventional ML-MM which may be particularly advantageous for considering missing or incomplete data scenarios. Other approaches used for estimating mixing proportions under partial or complete lack of nursery-source data have been based upon unsupervised clustering followed by discriminant analysis (Arkhipkin, Schuchert & Danyushevsky, 2009; Shima & Swearer, 2009; Schuchert, Arkhipkin & Koenig, 2010). Nonetheless, no independent assessments of bias or uncertainty seem to be available for this clustering approaches when applied to mixed stock analysis under incomplete sampling scenarios.

The large differences in chemical nursery signatures we found among cohorts in this dataset reflected large interanual variability in these nursery habitats (Tournois et al., 2013), which may be common to most shallow water and estuarine nursery areas (Secor, 2015). The high sensibility of ML-MM reliability to this inter-annual variability highlights the need to assure true independence among individual samples being used to build nursery-signature parameter estimates. Otherwise, variability among nursery-sources might be easily confounded with variability among years, schools, sampling events or other sources of correlation, commonly neglected in fisheries and ecological studies (Zuur, Ieno & Smith, 2007). Adding the effects of these random sources of correlation through a mixed or hierarchical approach (Bolker et al., 2009) has been already explored as extension of the EM algorithm by Heinzl & Tutz (2013). A very intuitive step here would be to combine data from multiple cohorts to improve the estimation of *K*, although this task could be also achieved using the joint likelihood from individual ML-MM models fit to each yearly dataset.

It is important to acknowledge that we did not explore sample size effects, but used constant sample sizes of 25 individuals per known nursery-source and 100 individuals per mixed-stock and cohort. While no major changes in bias would be expected at different sample sizes, an obvious reduction in uncertainty would be expected if the number of fish included in the nursery-source and/or mixed-stock datasets were larger. It would be of particular interest to consider intermediate scenarios where some small level of sampling existed for some/all nursery-sources, which maybe a common real-life situation when juvenile sampling is rather opportunistic. Under the unconditional approach we have followed, these pieces of information could be used without the risk of giving them full weight, as it would occur if they were used for producing fixed nursery-signature parameters, under the conditional approach. Moreover, these data, although limited, could be used to sustain some resampling procedures aimed to explore the risk of biased conclusions under plausible scenarios, as done in the present work.

In conclusion, unconditional ML-MM showed to be a suitable tool for estimating mixing proportions and nursery signatures of *S. aurata* under multiple scenarios that included incomplete sampling and a range of chemical signature separations among nursery-sources. In contrast, our approach yielded rather discouraging results regarding the estimation of the true number of nursery-sources (*K*), under incomplete sampling and/or identification of nursery-sources. Therefore, new efforts aimed to develop new mixed-stock analysis tools and/or to evaluate the performance of the existing ones are required. Such evaluations should be conducted over the widest possible range of species, habitats and biological scenarios, both to improve the reliability of these tools and to enhance our understanding about the structure and connectivity of exploited and threatened fish populations.

## Supplemental Information

10.7717/peerj.2415/supp-1 Supplemental Information 1 Methodological details.

10.7717/peerj.2415/supp-2 Supplemental Information 2 Descriptive statistics.

10.7717/peerj.2415/supp-3 Supplemental Information 3 Appendix 3. Main analyses (R script in pdf format).

10.7717/peerj.2415/supp-4 Supplemental Information 4 Elemental fingerprints data.

## Additional Information and Declarations

### Data Deposition

The following information was supplied regarding data availability: Niklitschek, Edwin (2016): three_years_data_elemental_ratio.csv. Figshare: https://dx.doi.org/10.6084/m9.figshare.2067789.v1.
