# Supplementary material for: Performance of maximum likelihood mixture models to estimate nursery habitat contributions to fish stocks: a case study on sea bream Sparus aurata
Source: PeerJ. 2016 Oct 4;4:e2415. doi: 10.7717/peerj.2415 (PMC5068389; doi:10.7717/peerj.2415)
Supplement: Supplemental Information 3 — Main analyses (R script in pdf format). [file peerj-04-2415-s003.pdf]

```
#-------------------------------------------------------------------------------------
## Created EN 20160106
## Resampling and FDM model fitting procedures using the EM algorithm on Sparus aurata
#-------------------------------------------------------------------------------------
rm(list=ls())
setwd("~/Sparus_2013"); getwd()
library(Matrix); library(gtools);library(vegclust)
library(mvnormtest);library(mvtnorm);library(MVN);
library(car);library(MASS);library(Matrix)
library(doParallel);library(foreach);
# Paralell computing set up
cl<-2; # Number of available cores for parallel computing
registerDoParallel(cl);getDoParWorkers()
nrep<-cl
#-------------------------------
# Basic set up
#-------------------------------
k2test=0:4; # Number of known sources to simulate
kmin=1; # Minimum number of sources to evaluate (should be >=known)
kmax=8; # Maximum number of sources to evaluate
yr=4; # Number of years to included in the analysis
by.year=TRUE; # If false, it pools data from all years in a single analysis
bl.fix=FALSE; # If true, conditional fit; if falase, unconditional fit
p=c(0.1,0.2,0.3,0.4); # mixing proportions in the mixed-stock sample
n.y=100 # sample size per year
sel.var<-c("Sr","Y","Ba","B","Li","Rb","Mg") # Tournois et al (2013)'s ordered selection I (solution)
#--------------------------------------------------
#1. Data manipulation
#------------------------------------------------
#1. Read data
excl<-c(212,223,274) # evident outliers to exclude
elements<- read.table("datos/three_years_data_elemental_ratio.csv",head=TRUE,sep=";",skip=1)[-excl,]
elements$Year=factor(elements$Year)
#-----------------------------------------------
# Test normality and performs transformations
#------------------------------------------------
lcu<-powerTransform(as.matrix(elements[,sel.var])~elements$Habitat*elements$Year,family="bcPower")$lambda
elements3<-elements
elements3[,sel.var]=scale(bcPower(elements[,sel.var],lcu))
nsel.var=length(sel.var)

# manova1<-manova(as.matrix(elements3[,sel.var])~elements3$Habitat*elements3$Year)
# r1<-resid(manova1)
# roystonTest(r1,qqplot=T)
# uniNorm(r1)
#------------------------------------------------------------------------------------------
# Resampling and modeling routines
#------------------------------------------------------------------------------------------
for (n.known in k2test)
{
  #Start the clock
  ptm<-proc.time()
  #define total number of origins to test so K>=KS and K=KS+KU
  if(n.known>=1) {
    if (kmin>=n.known){
      origins2test<-c(kmin:kmax)
    } else {origins2test<-c(n.known:kmax)}
  }else origins2test<-c(1:kmax)

  out2<-foreach (i=1:nrep,.combine=rbind,.errorhandling = "remove") %dopar%
  {
    #-------------------------------------------------------------------------------------
    # 1. Bootstrapping
    #-------------------------------------------------------------------------------------
    {
      #select year
      if(by.year==TRUE & yr<3){years<-sample(unique(elements3$Year),yr)} else {years=unique(elements3$Year)}
      years=years[order(years)]
      # split vailable observations by year and habitat
      list1<-split((1:nrow(elements3))[elements3$Year %in% years],elements3[elements3$Year %in%
years,c("Habitat","Year")],drop=T)
      # produce nursery-source (training) samples for all sources
      sel<-do.call("rbind",lapply(list1,function(x) {
        mu=colMeans(elements3[x,sel.var])
        vcov=cov(elements3[x,sel.var])
        data.frame(Individuals=1:25,mvrnorm(25,mu=mu,Sigma = vcov),
                   Habitat=unique(elements3$Habitat[x]),Year=unique(elements3$Year[x]))
      }))
      # Select and order known sources to be included in nursery-source dataset
      origin.sel<-sample(unique(elements3$Habitat),n.known);
      origin.sel=origin.sel[order(origin.sel)]
      # exclude unknown sources
      if(n.known>0) {
        known.data<-sel[sel$Habitat %in% origin.sel,]
      } else {known.data<-NULL}
      #---
      # produce mixed-stock data
      p.r=data.frame(origin=unique(elements3$Habitat),sample(p)) # Proportions in mixed-stock data
      new.data<-do.call("rbind",lapply(years,function(i) {
        do.call("rbind",lapply(1:4,function(j) {
```

```
      e.ij=elements3[elements3$Year==i & elements3$Habitat==unique(elements3$Habitat)[j],sel.var]
      mu=colMeans(e.ij);vcov=cov(e.ij)
      n.ij=n.y*p.r[j,2]
      data.frame(Individuals=1:n.ij,mvrnorm(n.ij,mu=mu,Sigma = vcov),
                 Habitat=unique(elements3$Habitat)[j],Year=i)
    }))
  }))
  if(by.year==FALSE) {new.data$Year="all";known.data$Year="all"}
  known.origin<-known.data$Habitat
}
#-------------------------------------------------------------------------------------
# 2. Starting mean and covariance parameters for known sources
#-------------------------------------------------------------------------------------
if (n.known>0)
{
  if (length(unique(known.data$Habitat))>0){
    if(by.year==TRUE){
      sp1<-split(known.data,known.data$Year)}else{
        sp1<-list(known.data)}
    mu.known=lapply(sp1,function(x){
      sp2<-split(x,factor(x$Habitat))
      lapply(sp2,function(y) colMeans(y[,sel.var]))
    })
    vcov.known=lapply(sp1,function(x){
      sp2<-split(x,factor(x$Habitat))
      lapply(sp2,function(y) cov(y[,sel.var]))
    })
  }
}
#-------------------------------------------------------------------------------------
# 3. Semi-supervised clustering
#-------------------------------------------------------------------------------------
{# Fits a semi-supervised cluster model
  if(by.year==TRUE) years.hs=years else years.hs="all"
  hs<-lapply(origins2test[origins2test!=n.known], function(k) {
    hs03<-lapply(years.hs, function(j) {
      hs02=new.data[new.data$Year==j,]
      if(n.known>0){
        vc=vegclust(hs02[,sel.var], mobileCenters=(k-n.known),nstart=100,
                    fixedCenters = do.call("rbind",mu.known[[paste(j)]]),method="KM")
        origin0=names(vc$memb)[apply(vc$memb,1,which.max)]
      }else{
        vc=vegclust(hs02[,sel.var], mobileCenters=k,nstart=100,method="KM")
        origin0=names(vc$memb)[apply(vc$memb,1,which.max)]
      }
      if(!is.null(origin0)){
        origin01<-c(as.numeric(substr(origin0,2,2))-(k-n.known))
        hs02$origin=NA
        hs02$origin[substr(origin0,1,1)=="M"]=origin0[substr(origin0,1,1)=="M"]
        if(n.known>0){hs02$origin[is.na(hs02$origin)]=names(mu.known[[1]])[origin01[substr(origin0,1,1)!="M"]]}
      }
      hs02})
    names(hs03)<-as.character(years.hs)
    hs04<-do.call(rbind,hs03)
    hs04
  })
  if (length(hs)>0){
    names(hs)<-origins2test[origins2test!=n.known]
    datamix<-hs[[1]]
  }else{datamix=new.data}
}
#-------------------------------------------------------------------------------------
# 4. Produce list of starting parameters
#-------------------------------------------------------------------------------------
{
  ipar.lst<-lapply(origins2test,function(s){
    b.list<-fpar<-ipar<-list()
    origin.list=c(1:s)
    #-------------------------------------------------------------------------------------
    # 4.1. Starting mean and covariance parameters
    #-------------------------------------------------------------------------------------
    # Update mu and cov for a mix known and unknown
    if(s>n.known)
    {
      test.data2=hs[[paste(s)]]
      #Means and covariances
      if (length(unique(test.data2$origin))>1){
        if(by.year==TRUE){
          sp1<-split(test.data2,test.data2$Year)}else{
            sp1<-list(test.data2)}
        mu.new=lapply(sp1,function(x){
          sp2<-split(x,x$origin)
          lapply(sp2,function(y) colMeans(y[,sel.var]))
        })
        vcov.new=lapply(sp1,function(x){
          sp2<-split(x,x$origin)
          lapply(sp2,function(y) cov(y[,sel.var]))
        })
      }else{
        if(by.year==TRUE){
          sp1<-split(test.data2,test.data2$Year)}else{
```

```
            sp1<-list(test.data2)}
          mu.new=lapply(sp1,function(x) colMeans(x[,sel.var]))
          vcov.new=lapply(sp1,function(x){
            cov(x[,sel.var])})
        }
      }
      #------------------------------------------------------------------------------------
      # 4.2. Starting mean and covariance parameters
      #------------------------------------------------------------------------------------
      if(s==n.known){
        mu.all=mu.known;vcov.all=vcov.known;
      }else{
        if(n.known==0){mu.all=mu.new;vcov.all=vcov.new}else{
          vcov.all<-lapply(as.character(years),function(y){
            l1=c(vcov.known[[y]],vcov.new[[y]][!(names(vcov.new[[y]]) %in% origin.sel)])
          });
          names(vcov.all)=years
          mu.all<-lapply(as.character(years),function(y){
            l1=c(mu.known[[y]],mu.new[[y]][!(names(mu.new[[y]]) %in% origin.sel)])
          }); names(mu.all)=years
        }
      }
      #Ensure all covariance matrices are positive definite
      vcov.all<-lapply(names(vcov.all),function(i)
      {
        if(s>1){
          aux2<-lapply(vcov.all[[i]], function(j) {
            if(!is.na(det(j))){if(det(j)<=1E-9){
              aux1<-cov(new.data[new.data$Year==i,sel.var])} else {
                aux1=j}} else {aux1<-cov(new.data[,sel.var])}
            names(aux1)=names(j)
            aux1
          })
        }else
        {
          if(n.known==0) j=vcov.all[[i]] else j=vcov.all[[i]][[1]]
          if(!is.na(det(j))){if(det(j)<=1E-9){
            aux1<-cov(test.data2[test.data2$Year==i,sel.var])} else {
              aux1=j}} else {aux1<-cov(test.data2[,sel.var])}
          names(aux1)=names(j)
          aux2=aux1
        }
      })
      names(vcov.all)=names(mu.all)
      ipar<-list(mu.all=mu.all,vcov.all=vcov.all)
      #------------------------------------------------------------------------------------
      # 4.3. Starting values for mixing proportions
      #------------------------------------------------------------------------------------
      # values are re-scaled logit (needed for other maximization algorithms although not for EM)
      if(s>1){
        if (by.year==TRUE)
        {
          pred<-data.frame()
          for (j in years){
            new.data.y=new.data[new.data$Year==j,]
            i.names=names(vcov.all[[paste(j)]])
            p.i=sapply(i.names,function(i){
              dmvnorm(as.matrix(new.data.y[,sel.var]),mu.all[[paste(j)]][[i]],vcov.all[[paste(j)]][[i]])
            })
            pred0<-data.frame(Year=j,pred=colnames(p.i)[apply(p.i,1,which.max)])
            pred<-rbind(pred,pred0)
          }
          if(s>n.known) {pred=hs[[paste(s)]][,c("Year","origin")];names(pred)[2]="pred"}
          for (y in 1:length(years))
          {
            datamix$mixture=1
            (p.mat<-table(datamix$mixture[datamix$Year==years[y]],pred$pred[pred$Year==years[y]],useNA = "ifany"))
            p.mat2<-(as.matrix(rbind(p.mat/rowSums(p.mat))))
            p.mat2<-ifelse(is.na(p.mat2),0.001,ifelse(p.mat2==1,1-(s-1)/1000,ifelse(p.mat2==0,0.001,p.mat2)))
#avoid log(0)
            p.mat2<-p.mat2/rowSums(p.mat2)
            b.list[[y]]<-log((p.mat2)/(1-p.mat2))[,1:(s)]
            names(b.list)[[y]]=paste(years[y])
          }
        }else{
          pred<-data.frame()
          p.i<-matrix(NA,nc=s,nr=nrow(new.data))
          for (i in 1:s){
            i.names=names(vcov.all[[1]])
            p.i[,i]<-dmvnorm(as.matrix(new.data[,sel.var]),mu.all[[1]][[i.names[[i]]]],vcov.all[[1]]
[[i.names[[i]]]])
          }
          pred0<-data.frame(Year="all",pred=names(mu.all[[1]])[apply(p.i,1,which.max)])
          pred<-rbind(pred,pred0)
          if(s>n.known) {pred=hs[[paste(s)]][,c("Year","origin")];names(pred)[2]="pred"}
          datamix$mixture=1
          (p.mat<-table(datamix$mixture,pred$pred,useNA = "ifany"))
          p.mat2<-(as.matrix(rbind(p.mat/rowSums(p.mat))))
          p.mat2<-ifelse(is.na(p.mat2),0.001,ifelse(p.mat2==1,1-(s-1)/1000,ifelse(p.mat2==0,0.001,p.mat2)))  #avoid
log(0)
          p.mat2<-p.mat2/rowSums(p.mat2)
```

```
      b.list[[1]]<-log((p.mat2)/(1-p.mat2))
      names(b.list)[[1]]="all"
    }
    {ipar[["b.list"]]=b.list}
  }
  if (s>1) {ipar[["b.list"]]=b.list}
  #-----------------------------------------------------------------------------------------
  # 4.4. Compilation of all starting parameter, data and other useful values
  #-----------------------------------------------------------------------------------------
  list(ipar=ipar,datamix=datamix[,c(sel.var,"Year")],origin.sel=origin.sel,year.sel=years)
 })
 names(ipar.lst)=origins2test
}
#-----------------------------------------------------------------------------------------
# Likelihood maximization through the EM algorithm
#-----------------------------------------------------------------------------------------
out1=lapply(ipar.lst,function(x)
{
 if(by.year==TRUE) years2=x$year.sel else years2="all"
 em.year=lapply(years2,function(y)
 {
  # Data matrix
  X=datamix;X=X[X$Year==y,sel.var]
  # Reconstruct parameter vectors and matrices (heritage from optim)
  known.origins=x$origin.sel
  pi=if(is.null(x$ipar$b.list)) 1 else 1/(1+exp(-x$ipar$b.list[[paste(y)]]))
  mu=x$ipar$mu.all[[paste(y)]];mu=mu[order(names(mu))]
  sigma=x$ipar$vcov.all[[paste(y)]]
  if(length(pi)>1){sigma<-sigma[order(names(sigma))]}
  # Working starting values
  mu.em=mu;pi.em=pi;sigma.em=sigma;nsources=length(pi)
  # Produces joint dataset
  XK=known.data[known.data$Year==y,]
  if(n.known>0){X2=rbind(X,XK[,sel.var])}else{X2=X}
  # Likelihood maximization for K>1
  if(length(pi)>1)
  {
   #Compute starting likelihood
   loglik<- vector()
   loglik[1]<-0
   ld0=sapply(names(mu),function(i){
    mvtnorm::dmvnorm(as.matrix(X),mu.em[[i]],sigma.em[[i]])
   })
   loglik[2]<-sum(log(rowSums(t(apply(ld0,1,function(j) pi.em*j)))))
   k=2
   # EM algorithm
   while(abs(loglik[k]-loglik[k-1])>=1E-7 & k<=300) {
    # E step
    tau.source=sapply(names(mu),function(i){
     (XK$Habitat==i)*1
    })
    dd=sapply(names(sigma),function(i){
     dmvnorm(as.matrix(X),mu.em[[i]],sigma.em[[i]])
    })
    tau.mixed<-t(apply(dd,1,function(j) j*pi.em/sum(j*pi.em)))
    # Add source data
    if(n.known>0){tau=rbind(tau.mixed,tau.source)}else{tau=tau.mixed}
    if(ncol(dd)==1) tau=t(tau)
    colnames(tau)=names(mu)
    tau[is.na(tau)]=1/nsources
    # M step
    pi.em<-apply(tau.mixed,2,function(i) sum(i)/length(i))
    if(bl.fix==FALSE){# Performs Unconditional estimation of mu and theta for all nursery-sources
     # Compute updated mu values for all sources
     mu.em<-split(t(apply(tau,2,function(i) colSums(i*X2)/sum(i))),names(mu.em))
     # rename
     mu.em<-lapply(mu.em,function(i) {j=matrix(i,nr=1);colnames(j)=sel.var;j})
     # Compute updated sigma values for all sources
     sigma.em<-lapply(names(sigma.em),function(i) {
      res.i<-t(apply(X2,1,function(x) x-mu.em[[i]]))
      cov.i=cov.wt(res.i, wt=tau[,i])$cov
      if(is.finite(det(cov.i)) & det(cov.i)>1e-9) cov.i else sigma.em[[i]]
     })
     # rename
     names(sigma.em)=names(sigma)
    }else{# Limits unconditional estimation of mu and theta to unknown nursery-sources
     # Compute updated mu values for all sources
     mu.em0<-split(t(apply(tau,2,function(i) colSums(i*X)/sum(i))),names(mu.em))
     # rename
     mu.em0<-lapply(mu.em0,function(i) {j=matrix(i,nr=1);colnames(j)=sel.var;j})
     # Pass along updated mu values only for unknown origins
     mu.em=c(mu[names(mu) %in% known.origins],mu.em0[!(names(mu.em0) %in% known.origins)])
     mu.em=mu.em[order(names(mu.em))]
     # Compute updated sigma values for all sources
     sigma.em0<-lapply(names(sigma),function(i) {
      res.i<-t(apply(X,1,function(x) x-mu.em[[i]]))
      cov.i=cov.wt(res.i, wt=tau[,i])$cov
      if(is.finite(det(cov.i)) & det(cov.i)>1e-9) cov.i else sigma.em[[i]]
     })
     names(sigma.em0)=names(sigma)
     # Pass along updated sigma values only for unknown origins
```

```
          sigma.em=c(sigma[names(sigma) %in% known.origins],sigma.em0[!(names(sigma.em0) %in% known.origins)])
          sigma.em=sigma.em[order(names(sigma.em))]
        }
        #probability density given updated parameters
        ld.test=sapply(names(mu),function(i){
          mvtnorm::dmvnorm(as.matrix(X2),mu.em[[i]],sigma.em[[i]],log=T)
        })
        #log-likelihood given updated parameters
        if(ncol(ld.test)>1){
          loglik[k+1]<-sum(colSums(tau*(t(apply(ld.test,1,function(x) log(pi.em)+x)))))
        }else{
          loglik[k+1]<-sum(colSums(tau*(apply(ld.test,1,function(x) log(pi.em)+x))))
        }
        k<-k+1
      }
      if(k>=300){loglik<-9999}

      #Final probability densities
      ld.fin=sapply(names(mu.em),function(i){
        mvtnorm::dmvnorm(as.matrix(X),mu.em[[i]],sigma.em[[i]])
      })
      tau.fin<-t(apply(ld.fin,1,function(j) j*pi.em/sum(j*pi.em)))
    }
    #Final Likelihood and mixing proportions
    if(length(pi)>1) {
      pi.fin<-pi.em
      loglik.fin<-sum(log(rowSums(t(apply(ld.fin,1,function(x) pi.fin*x)))))
    } else {
      pi.fin=1
      if(n.known==1) mu=mu[[1]]
      loglik.fin<-sum(log(mvtnorm::dmvnorm(as.matrix(X),mu,sigma)))
    }
    # Rename unknown groups by maximizing correct classification (only for 4-source models)
    if(length(mu.em)==4){
      cc0=data.frame(Habitat=new.data$Habitat[new.data$Year==y]
                     ,ph=colnames(ld.fin)[apply(ld.fin,1, which.max)])
      cc1=as.data.frame(cbind(table(cc0$ph,cc0$Habitat)))
      #select combination that maximize correct assignations
      perm=permutations(4,4)
      perm.sum=sapply(1:nrow(perm),function(j){
        cc1[1,perm[j,1]]+cc1[2,perm[j,2]]+cc1[3,perm[j,3]]+cc1[4,perm[j,4]]
      })
      names(mu.em)=unique(new.data$Habitat)[perm[which.max(perm.sum),]]
    }
    #Output list
    list(mu=mu.em,pi=pi.fin,sigma=sigma.em,loglik=loglik.fin,nx=nrow(X),nsources=nsources)
  })
  names(em.year)=years2
  em.year
})
#Prepare output 2
if (!is.null(out1)){
  names(out1)=origins2test
  #AIC
  AICs<-matrix(sapply(out1,function(i) {sapply(i, function(j){
    ifelse(bl.fix==TRUE,-2*j$loglik+2*(j$nsources+(j$nsources-n.known)*(7*2+(49-7)/2)-1),
           -2*j$loglik+2*(j$nsources*(1+7*2+(49-7)/2)-1))
  })}),nc=length(ipar.lst))
  #BIC
  BICs<-matrix(sapply(out1,function(i) {sapply(i, function(j){
    ifelse(bl.fix==TRUE,-2*j$loglik+log(j$nx)*(j$nsources+(j$nsources-n.known)*(7*2+(49-7)/2)-1),
           -2*j$loglik+log(j$nx)*(j$nsources*(1+7*2+(49-7)/2)-1))
  })}),nc=length(ipar.lst))
  #Several values of interest
  loglik<-matrix(sapply(out1,function(i) sapply(i, function(j) j$loglik)),nc=length(ipar.lst))
  best.model.aic=names(ipar.lst)[apply(AICs,1,which.min)]
  best.aic=apply(AICs,1,function(i) i[which.min(i)])
  best.model.bic=names(ipar.lst)[apply(BICs,1,which.min)]
  best.bic=apply(BICs,1,function(i) i[which.min(i)])
  bic.dist=apply(BICs,1,function(i) (min(i)-mean(i))/sd(i))
  #Defines output data-frame
  out1.2=data.frame(by.year=by.year,bl.fix=bl.fix,n.known=n.known,run=i,
                    Year=rep(names(out1[[1]]),each=4),
                    origin=unlist(lapply(out1[["4"]],function(j) names(j[["mu"]]))),
                    source.data=unlist(lapply(out1[["4"]],function(k) names(k[["sigma"]]))),
                    do.call("rbind",lapply(out1[["4"]],function(l) {do.call("rbind",l[["mu"]])})),
                    known.origins=paste(ipar.lst[["4"]]$origin.sel,collapse="-"),
                    known.years=paste(ipar.lst[["4"]]$year.sel,collapse="-"),
                    best.model.aic=rep(best.model.aic,each=4),
                    best.aic=rep(best.aic,each=4),
                    best.model.bic=rep(best.model.bic,each=4),
                    best.bic=rep(best.bic,each=4),bic.dist=rep(bic.dist,each=4),
                    pred.mix=unlist(lapply(out1[["4"]],function(l) l[["pi"]])))
  # Add true mixing proportion in mixed-stock data
  out1.2=merge(out1.2,p.r,by="origin")
  out1.2=out1.2[order(out1.2$Year,out1.2$origin),]
}else {out1.2=NULL}
out1.2
}
out2    # Stop the clock
elapsed=round((proc.time()-ptm)[3]/3600,2)
```

```
  # Write the output
  write.csv(out2,paste("EM known
",n.known,".by.year_",by.year,".bl.fix_",bl.fix,".",Sys.time(),elapsed,".",".csv",sep=""),row.names=F)
}
```
