# Supplementary material for: Performance of maximum likelihood mixture models to estimate nursery habitat contributions to fish stocks: a case study on sea bream Sparus aurata
Source: PeerJ. 2016 Oct 4;4:e2415. doi: 10.7717/peerj.2415 (PMC5068389; doi:10.7717/peerj.2415)
Supplement: Supplementary file 4 [file peerj-04-2415-s001.odt]

Appendix 1

Resampling and estimation procedures

- Define the range of the total number of nursery-sources to be evaluated (K=KMIN:KMAX), and the number of nursery-sources to be treated as known and previously sampled for baselines (KS=0-4).

- Normalize data using a multivariate version of the Box-Cox transformation (Fox & Weisberg, 2011), considering potential effects of cohort, nursery-source and their interaction. 

- Standardize all (transformed) chemical compositions to TexMaths12§display§\bar x§svg§600§FALSE§=0 and TexMaths12§display§tr (\bold {S})§svg§600§FALSE§=1

Within each cohort:

- Use parametric bootstrapping to produce the following two datasets:

  - A nursery-source dataset containing 25 random samples from each of the KS nursery-sources selected randomly as “known” sources. 

  - A mixed-stock dataset containing 100 random samples from all four nursery-sources, mixed in, randomly allocated, proportions of 0.1, 0.2, 0.3 and 0.4.

- Compute starting TexMaths12§display§\hat\theta_{k}§svg§600§FALSE§ parameters (TexMaths12§display§\hat\mu_{k}§svg§600§FALSE§, TexMaths12§display§\hat\Sigma_{k}§svg§600§FALSE§) for each of the (KS≤4) known nursery-sources included in the nursery-source dataset.

- Input TexMaths12§display§\hat\mu_{k}§svg§600§FALSE§ estimates from step 5, as fixed centroids, into the semi-supervised K-means clustering algorithm implemented in the R-package veganclust (De Cáceres, Font & Oliva, 2010). Allow the clustering model to estimate additional (mobile) centroids (TexMaths12§display§\hat\mu_{k}§svg§600§FALSE§) for all KU unknown sources, and apply it to cluster all mixed-stock data. 

- Use the mixed-stock data clustered into the KU  new clusters (step 5) to estimate additional TexMaths12§display§\hat\theta_{k}§svg§600§FALSE§ parameters for all unknown nursery-sources.

- Use all TexMaths12§display§\hat\theta_{k}§svg§600§FALSE§ parameters (from steps and 5 and 7), assume TexMaths12§display§x_{ik}§svg§600§FALSE§~MVN(TexMaths12§display§\hat\theta_{k}§svg§600§FALSE§) and assign each mixed-data observation (TexMaths12§display§x_{ik}§svg§600§FALSE§) to the putative nursery-source k that maximizes TexMaths12§display§p(x_{ik}|\hat\theta_k)§svg§600§FALSE§.

- Compute starting TexMaths12§display§\hat p_{k}§svg§600§FALSE§ values from the proportions of mixed-stock data allocated to each putative nursery-source (k) at step 6.

- Initiate the EM algorithm (see next section) using starting parameters (TexMaths12§display§\hat\theta_{k}§svg§600§FALSE§ and TexMaths12§display§\hat p_{jk}§svg§600§FALSE§) obtained at steps 4, 6 and 8, and iterate it until likelihood improvement becomes lower than 1E-7.

- For selecting the most informative value of K, repeat steps 6 to 10 for all K values between KS and KMAX, compute Schwarz's (1978) Bayesian Information Criteria for each model, and select the one exhibiting the lowest BIC.   

- Repeat  steps 1 to 11 and save outputs for r=1000 resampling runs. 

Expectation-Maximization algorithm, modified from Dempster, Laird & Rubin (1977)

E-Step

TexMaths12§display§ \hat\tau_{ik}={{\hat p_k \cdot g(x_i|\hat\theta_k)+z_k \cdot g(y_i|\hat\theta_k)}
\over
{\sum_{k=1}^K{\hat p_k \cdot g(x_i|\hat\theta_k)}}+n_y}§svg§600§FALSE§

M-Step

TexMaths12§display§\hat p_k^{new}={{\hat p_k \cdot g(x_i|\hat\theta_k)}
\over
{n_k \cdot \sum_{k=1}^K{\hat p_k \cdot g(x_i|\hat\theta_k)}}}§svg§600§FALSE§

TexMaths12§display§\hat \mu_k^{new}={ {\sum_{i=1}^{N}\hat \tau_{ik} \cdot x_i} \over {\sum_{i=1}^{N}\hat \tau_{ik}}}§svg§600§FALSE§

TexMaths12§display§\hat \Sigma_k^{new}={ {\sum_{i=1}^{N}\hat \tau_{ik} \cdot
(x_i-\hat \mu_k^{new})(x_i-\hat \mu_k^{new})^T} 
\over {\sum_{i=1}^{N}\hat \tau_{ik}}}§svg§600§FALSE§

BIC

TexMaths12§display§BIC=-2LL_K+log_e(n_x+n_y)\cdot np_K§svg§600§FALSE§

where,

TexMaths12§display§\hat p_k§svg§600§FALSE§=estimated mixing proportion of nursery-source k

TexMaths12§display§z_k§svg§600§FALSE§=indicator variable, TexMaths12§display§z_k§svg§600§FALSE§=1 when TexMaths12§display§y_i§svg§600§FALSE§ was sampled at nursery-source; TexMaths12§display§z_k§svg§600§FALSE§=0, otherwise

TexMaths12§display§x_i§svg§600§FALSE§,TexMaths12§display§y_i§svg§600§FALSE§=elemental composition observations at mixed-stock and nursery-source datasets, respectively

TexMaths12§display§n_i§svg§600§FALSE§,TexMaths12§display§n_k§svg§600§FALSE§=number of observations at the mixed-stock and nursery-source datasets, respectively

TexMaths12§display§\hat\theta_k§svg§600§FALSE§=estimated baselines (TexMaths12§display§\hat\mu_k§svg§600§FALSE§,TexMaths12§display§\hat\Sigma_k§svg§600§FALSE§) for nursery k

TexMaths12§display§\hat\mu_k§svg§600§FALSE§=estimated vector of mean elemental compositions for source k

TexMaths12§display§\hat\Sigma_k§svg§600§FALSE§=estimated covariance matrix for elemental compositions at source k

TexMaths12§display§K§svg§600§FALSE§=total number of nursery-sources contributing to the mixed-stock

TexMaths12§display§LL_K§svg§600§FALSE§=loge-likelihood of the TexMaths12§display§K§svg§600§FALSE§ nursery-sources model

TexMaths12§display§np_K§svg§600§FALSE§=number of parameters estimated to fit the TexMaths12§display§K§svg§600§FALSE§ nursery-sources model

References

De Cáceres M., Font X., Oliva F. 2010. The management of vegetation classifications with fuzzy clustering. Journal of Vegetation Science 21:1138–1151.

Dempster AP., Laird NM., Rubin DB. 1977. Maximum likelihood from incomplete data via the EM algorithm. Journal of the Royal Statistical Society. Series B:1–38.

Fox J., Weisberg S. 2011. An R companion to applied regression. California, USA.: Sage Publications.

Schwarz G. 1978. Estimating the dimension of a model. Annals of Statistics 6:461–464.
