# Supplementary material for: Performance of maximum likelihood mixture models to estimate nursery habitat contributions to fish stocks: a case study on sea bream Sparus aurata
Source: PeerJ. 2016 Oct 4;4:e2415. doi: 10.7717/peerj.2415 (PMC5068389; doi:10.7717/peerj.2415)
Supplement: Supplementary file 5 [file peerj-04-2415-s002.odt]

2008

Vector of means

| Habitat | Sr | Y | Ba | B | Li | Rb | Mg |
| --- | --- | --- | --- | --- | --- | --- | --- |
| Bages-Sigean | 5.24 | 29.75 | 15.86 | 1.2 | 0.55 | 84.69 | 48.94 |
| Mauguio | 4.76 | 23.39 | 14.42 | 1.21 | 0.34 | 106.68 | 54 |
| Salses-Leucate | 6.13 | 30.17 | 10.1 | 2.39 | 0.67 | 106.2 | 50.1 |
| Thau | 5.89 | 44.06 | 13.1 | 2.04 | 0.74 | 96.85 | 47.16 |

Covariance matrices 

|  | Sr | Y | Ba | B | Li | Rb | Mg |
| --- | --- | --- | --- | --- | --- | --- | --- |
| Bages-Sigean |
| Sr | 0.3428 | 0.2224 | 0.5595 | 0.1008 | 0.0364 | 4.5952 | -0.5476 |
| Y | 0.2224 | 16.4527 | -0.9644 | 0.4514 | -0.1496 | -23.7619 | 2.8342 |
| Ba | 0.5595 | -0.9644 | 7.439 | -0.188 | 0.0003 | 9.193 | 0.785 |
| B | 0.1008 | 0.4514 | -0.188 | 0.1692 | 0.0276 | 1.3376 | -0.1225 |
| Li | 0.0364 | -0.1496 | 0.0003 | 0.0276 | 0.0106 | 0.9278 | -0.0965 |
| Rb | 4.5952 | -23.7619 | 9.193 | 1.3376 | 0.9278 | 171.3983 | -17.3294 |
| Mg | -0.5476 | 2.8342 | 0.785 | -0.1225 | -0.0965 | -17.3294 | 17.4704 |

| Mauguio |
| --- |
| Sr | 0.2116 | 0.9604 | -0.4193 | 0.0954 | 0.0248 | -1.1206 | -1.443 |
| Y | 0.9604 | 6.3516 | -3.0406 | 0.62 | 0.1701 | -10.1751 | -7.1049 |
| Ba | -0.4193 | -3.0406 | 7.468 | -0.7648 | -0.1109 | 18.6845 | 6.4304 |
| B | 0.0954 | 0.62 | -0.7648 | 0.1619 | 0.0168 | -0.8091 | -1.2309 |
| Li | 0.0248 | 0.1701 | -0.1109 | 0.0168 | 0.0089 | -0.2372 | -0.0942 |
| Rb | -1.1206 | -10.1751 | 18.6845 | -0.8091 | -0.2372 | 234.7458 | 28.7817 |
| Mg | -1.443 | -7.1049 | 6.4304 | -1.2309 | -0.0942 | 28.7817 | 46.0696 |

| Salses-Leucate |
| --- |
| Sr | 0.3466 | 0.6622 | 0.601 | -0.0292 | -0.0068 | 4.0922 | -0.3374 |
| Y | 0.6622 | 6.06 | 0.8981 | 0.3797 | 0.0345 | 7.9977 | -0.9909 |
| Ba | 0.601 | 0.8981 | 9.1654 | -0.3281 | -0.0316 | 0.2666 | -1.6229 |
| B | -0.0292 | 0.3797 | -0.3281 | 0.1464 | 0.0026 | 0.498 | -0.9737 |
| Li | -0.0068 | 0.0345 | -0.0316 | 0.0026 | 0.0016 | 0.0175 | 0.0698 |
| Rb | 4.0922 | 7.9977 | 0.2666 | 0.498 | 0.0175 | 126.1887 | -0.6296 |
| Mg | -0.3374 | -0.9909 | -1.6229 | -0.9737 | 0.0698 | -0.6296 | 24.0509 |

| Thau |
| --- |
| Sr | 0.2497 | 1.6826 | -0.0394 | -0.005 | -0.004 | -1.1637 | -0.5061 |
| Y | 1.6826 | 13.6062 | -0.0835 | -0.0273 | 0.0199 | -6.6534 | -4.859 |
| Ba | -0.0394 | -0.0835 | 16.5906 | 0.5517 | 0.049 | -6.0478 | -1.249 |
| B | -0.005 | -0.0273 | 0.5517 | 0.1227 | -0.014 | -0.6002 | 0.7855 |
| Li | -0.004 | 0.0199 | 0.049 | -0.014 | 0.0078 | 0.3849 | -0.0069 |
| Rb | -1.1637 | -6.6534 | -6.0478 | -0.6002 | 0.3849 | 157.0355 | -25.0046 |
| Mg | -0.5061 | -4.859 | -1.249 | 0.7855 | -0.0069 | -25.0046 | 31.8386 |

2010

Vector of means

| Habitat | Sr | Y | Ba | B | Li | Rb | Mg |
| --- | --- | --- | --- | --- | --- | --- | --- |
| Bages-Sigean | 4.26 | 48.87 | 13.66 | 1.51 | 0.71 | 150.95 | 48.53 |
| Mauguio | 3.8 | 46.91 | 9.62 | 1.53 | 0.69 | 159.85 | 59.83 |
| Salses-Leucate | 5.47 | 37.4 | 18.58 | 1.82 | 0.69 | 154.47 | 52.82 |
| Thau | 6.45 | 43.89 | 9.11 | 1.4 | 0.61 | 134.23 | 44.23 |

Covariance matrices 

|  | Sr | Y | Ba | B | Li | Rb | Mg |
| --- | --- | --- | --- | --- | --- | --- | --- |
| Bages-Sigean |
| Sr | 0.1653 | 3.1074 | 0.8681 | 0.2419 | 0.0371 | 8.3658 | 1.6101 |
| Y | 3.1074 | 74.239 | 20.3052 | 4.7283 | 0.9853 | 210.1342 | 44.4423 |
| Ba | 0.8681 | 20.3052 | 14.7817 | 0.942 | 0.22 | 60.0064 | 6.5448 |
| B | 0.2419 | 4.7283 | 0.942 | 0.5171 | 0.0865 | 15.5003 | 3.4537 |
| Li | 0.0371 | 0.9853 | 0.22 | 0.0865 | 0.0234 | 3.4848 | 0.9125 |
| Rb | 8.3658 | 210.1342 | 60.0064 | 15.5003 | 3.4848 | 740.8735 | 161.1336 |
| Mg | 1.6101 | 44.4423 | 6.5448 | 3.4537 | 0.9125 | 161.1336 | 48.2752 |
| Mauguio |
| Sr | 0.1522 | 3.1904 | 0.4287 | 0.0523 | 0.0257 | 0.8334 | -0.8816 |
| Y | 3.1904 | 143.3651 | 15.505 | 1.5462 | 0.4118 | 157.3244 | -26.7568 |
| Ba | 0.4287 | 15.505 | 6.1441 | -0.0592 | -0.0375 | 17.3656 | -3.94 |
| B | 0.0523 | 1.5462 | -0.0592 | 0.1641 | 0.0459 | 4.5269 | 0.7062 |
| Li | 0.0257 | 0.4118 | -0.0375 | 0.0459 | 0.0172 | 0.8072 | 0.2831 |
| Rb | 0.8334 | 157.3244 | 17.3656 | 4.5269 | 0.8072 | 565.0074 | 58.7135 |
| Mg | -0.8816 | -26.7568 | -3.94 | 0.7062 | 0.2831 | 58.7135 | 81.3301 |
| Salses-Leucate |
| Sr | 0.7721 | 5.3713 | -1.3041 | 0.307 | 0.1158 | 6.1871 | 5.5361 |
| Y | 5.3713 | 106.7618 | -16.0362 | 3.5045 | 0.9459 | 43.1503 | 41.6309 |
| Ba | -1.3041 | -16.0362 | 64.5556 | -1.9087 | -0.5708 | 22.704 | -2.6854 |
| B | 0.307 | 3.5045 | -1.9087 | 0.2187 | 0.0625 | 3.3171 | 2.4388 |
| Li | 0.1158 | 0.9459 | -0.5708 | 0.0625 | 0.0232 | 1.1154 | 0.9927 |
| Rb | 6.1871 | 43.1503 | 22.704 | 3.3171 | 1.1154 | 548.6806 | 59.7686 |
| Mg | 5.5361 | 41.6309 | -2.6854 | 2.4388 | 0.9927 | 59.7686 | 72.0418 |
| Thau |
| Sr | 0.9693 | 5.0905 | 1.2654 | 0.1953 | 0.0874 | 20.468 | 4.9656 |
| Y | 5.0905 | 72.117 | 3.3673 | 2.651 | 0.6296 | 197.0761 | 27.798 |
| Ba | 1.2654 | 3.3673 | 6.9099 | -0.1488 | 0.0122 | 24.6987 | 2.8827 |
| B | 0.1953 | 2.651 | -0.1488 | 0.2386 | 0.0671 | 7.3719 | 3.1452 |
| Li | 0.0874 | 0.6296 | 0.0122 | 0.0671 | 0.0272 | 2.7736 | 1.4675 |
| Rb | 20.468 | 197.0761 | 24.6987 | 7.3719 | 2.7736 | 823.3886 | 170.6282 |
| Mg | 4.9656 | 27.798 | 2.8827 | 3.1452 | 1.4675 | 170.6282 | 97.9239 |

2011

Vector of means

| Habitat | Sr | Y | Ba | B | Li | Rb | Mg |
| --- | --- | --- | --- | --- | --- | --- | --- |
| Bages-Sigean | 5 | 25.81 | 12.07 | 1.31 | 0.51 | 89.89 | 46.63 |
| Mauguio | 4.37 | 23.82 | 10.62 | 1.09 | 0.44 | 96.87 | 51.48 |
| Salses-Leucate | 4.86 | 31.3 | 11.45 | 1.71 | 0.58 | 99.15 | 48.81 |
| Thau | 5.03 | 32.63 | 7.71 | 1.81 | 0.64 | 88.58 | 51.44 |

Covariance matrices: 

|  | Sr | Y | Ba | B | Li | Rb | Mg |
| --- | --- | --- | --- | --- | --- | --- | --- |
| Bages-Sigean |
| Sr | 0.2085 | 1.4539 | -0.2573 | 0.1263 | 0.0174 | 1.6436 | 0.0151 |
| Y | 1.4539 | 28.6297 | -4.0538 | 2.0152 | 0.4561 | 5.79 | 5.8723 |
| Ba | -0.2573 | -4.0538 | 4.8944 | -0.6058 | -0.1239 | 11.2244 | 1.8303 |
| B | 0.1263 | 2.0152 | -0.6058 | 0.194 | 0.0405 | -0.4154 | -0.0805 |
| Li | 0.0174 | 0.4561 | -0.1239 | 0.0405 | 0.0106 | -0.2949 | 0.0761 |
| Rb | 1.6436 | 5.79 | 11.2244 | -0.4154 | -0.2949 | 201.9955 | -0.5825 |
| Mg | 0.0151 | 5.8723 | 1.8303 | -0.0805 | 0.0761 | -0.5825 | 37.7747 |
| Mauguio |
| Sr | 0.3694 | 1.7905 | 0.0177 | 0.2303 | 0.0658 | -3.7105 | 0.6601 |
| Y | 1.7905 | 26.9789 | 0.7091 | 1.6022 | 0.4916 | 3.614 | 0.7072 |
| Ba | 0.0177 | 0.7091 | 6.5016 | 0.0737 | 0.0114 | 23.2813 | 0.418 |
| B | 0.2303 | 1.6022 | 0.0737 | 0.209 | 0.0567 | -2.9981 | -0.0083 |
| Li | 0.0658 | 0.4916 | 0.0114 | 0.0567 | 0.0177 | -1.0039 | -0.0461 |
| Rb | -3.7105 | 3.614 | 23.2813 | -2.9981 | -1.0039 | 284.9671 | 26.6176 |
| Mg | 0.6601 | 0.7072 | 0.418 | -0.0083 | -0.0461 | 26.6176 | 20.639 |
| Salses-Leucate |
| Sr | 0.1239 | -0.2496 | 0.0442 | -0.0183 | -0.0156 | -0.0646 | -0.2376 |
| Y | -0.2496 | 56.2718 | 3.091 | 3.6862 | 1.0018 | 63.3943 | 40.6249 |
| Ba | 0.0442 | 3.091 | 6.4539 | 0.0971 | 0.0252 | 0.3382 | 7.5433 |
| B | -0.0183 | 3.6862 | 0.0971 | 0.3625 | 0.0808 | 5.2777 | 2.3832 |
| Li | -0.0156 | 1.0018 | 0.0252 | 0.0808 | 0.0224 | 1.1665 | 0.7223 |
| Rb | -0.0646 | 63.3943 | 0.3382 | 5.2777 | 1.1665 | 250.2225 | 67.8461 |
| Mg | -0.2376 | 40.6249 | 7.5433 | 2.3832 | 0.7223 | 67.8461 | 67.0499 |
| Thau |
| Sr | 0.145 | -0.0586 | 0.2283 | -0.0002 | -0.0021 | -1.5519 | 0.2967 |
| Y | -0.0586 | 64.8037 | 6.9439 | 1.949 | 1.0001 | 98.573 | 41.0884 |
| Ba | 0.2283 | 6.9439 | 5.0535 | 0.1066 | 0.0703 | 12.5764 | 9.7794 |
| B | -0.0002 | 1.949 | 0.1066 | 0.1648 | 0.0441 | 2.5928 | 2.0083 |
| Li | -0.0021 | 1.0001 | 0.0703 | 0.0441 | 0.0195 | 1.4481 | 0.707 |
| Rb | -1.5519 | 98.573 | 12.5764 | 2.5928 | 1.4481 | 343.6469 | 118.0161 |
| Mg | 0.2967 | 41.0884 | 9.7794 | 2.0083 | 0.707 | 118.0161 | 121.5574 |
